# Metabolic Profiling of Human Peripheral Blood Mononuclear Cells: Influence of Vitamin D Status and Gender

**DOI:** 10.3390/metabo4020248

**Published:** 2014-04-22

**Authors:** Magdalena Stepien, Anne P. Nugent, Lorraine Brennan

**Affiliations:** 1 UCD Institute of Food and Health, University College Dublin, Belfield, Dublin 4, Ireland; E-Mails: stepienm@fellows.iarc.fr (M.S.); anne.nugent@ucd.ie (A.P.N.); 2 UCD Conway Institute, University College Dublin, Belfield, Dublin 4, Ireland

**Keywords:** peripheral blood mononuclear cells (PBMC), vitamin D, gender, metabolic profile

## Abstract

Metabolic profiling of peripheral blood mononuclear cells (PBMC) could serve as a less invasive and more direct alternative to tissue biopsies or serum in metabolomic research. We conducted two exploratory independent studies in order to characterise PBMC’s metabolomic profile following short-term vitamin D3 supplementation and to determine gender effects. In the first study, eight healthy males and females aged 40–65 y were randomly selected for profiling of PBMCs after receiving either 15 µg of vitamin D3 or placebo for four weeks. In the second study, twenty younger healthy males and females were studied. Cell metabolites were extracted and deproteinised using methanol/chloroform/water method and analysed by GC-MS. Higher vitamin D status had no effect on the fatty acid profile of PBMCs, but inflammatory biomarkers and adipokines correlated positively with stearic acid levels. In the second study, no gender-specific metabolites were identified. Valine, leucine and aspartic acid were identified as potential BMI-sensitive amino acids. Larger studies are needed to confirm the influence of BMI on these parameters. This work clearly demonstrates the utility of metabolomics profiling of PBMCs and paves the way for future applications of metabolomics in identifying metabolic profiles of blood cells as a measure for dietary intakes or physiological status.

## 1. Introduction

The “omics” techniques represent a powerful tool in modern biology by enabling high throughput measurements of many genes, proteins and metabolites in samples. Gene expression and, consequently, protein and metabolite levels may undergo changes in different physiological conditions. Within the “omics” field, metabolomics targets small molecules present in biological fluids or tissues [1]. In recent years, applications of metabolomics in nutrition research have increased. In general, applications can be broadly defined into three categories: (1) identification of novel biomarkers of dietary intake; (2) applications related to dietary interventions with the aim of enhancing the understanding of the effects of diets/foods on metabolic pathways; and (3) applications to diet-related diseases [2].

Metabolomics studies can be performed using several specimens [3]. The most easily obtainable are blood and urine samples. However, these do not reflect tissue metabolism, due to a transient presence of metabolites in the circulation. Tissue biopsies are difficult to obtain and often not acceptable to the study subjects. Several studies have championed the potential use of peripheral blood mononuclear cells (PBMC), a group of cells, including lymphocytes and monocytes/macrophages, in characterising gene expression patterns distinctive for certain diseases [4,5]. Transcriptomic profiling of PBMC following the ingestion of anti-inflammatory PUFA has been intensely investigated [6,7,8]; however, only a few studies reported the lipid profile of PBMCs [9,10,11]. Gene expression profiling of PBMCs has been successfully used to detect differences between dietary groups, where distinct differences in gene expression were found after consumption of PUFA and SFA [7]. Supplementation with fish oil for 26 weeks resulted in a less inflammatory profile of PBMC, i.e., decreased expression of genes involved in inflammatory and atherogenic pathways [12]. Furthermore, targeting the fatty acids (FAs) of PBMCs demonstrated that the intake of specific FA can be reflected in the FA profile of PBMC phospholipids [11,13]. Furthermore, the FA composition of PBMC phospholipids was related to differences in immune cell functions in healthy subjects [10]. Despite these positive applications of the targeted profiling of PBMCs, the approach is relatively under-used in nutrition research.

The health effects of vitamin D have recently been shown to extend beyond its classical bone function. Recent literature supports a potential anti-inflammatory function of vitamin D [14]. PBMCs express vitamin D receptors (VDR) and vitamin D is an inhibitor of T-cell cytokine production [15]. Moreover, sufficient vitamin D levels are negatively associated with plasma lipids, as a result of decreased triglycerides (TG) absorption or increased TG peripheral removal [16]. Therefore, PBMC profiling could reveal a specific anti-inflammatory profile related to elevated vitamin D intake.

The main objective of the present work was to demonstrate the application of metabolomic profiling of PBMCs. To achieve this, two exploratory studies were utilized: Study 1 examined the impact of vitamin D supplementation on the lipid profile of PBMCs, and Study 2 examined gender effects in the PBMC fatty acid and amino acid profiles.

## 2. Results and Discussion

### 2.1. Study 1: Effect of Supplementation with Vitamin D on PBMC Fatty Acid Levels

PBMC samples from subjects (N = 8) receiving a vitamin D supplement or a placebo were isolated and the FA profiled. The subjects in the supplemented and placebo groups did not significantly differ by age, BMI (Table 1), as well as dietary intakes of SFA, MUFA and PUFA.

In Study 1, several biochemical markers related to metabolic health and inflammation were measured. Circulating 25(OH)D levels increased significantly in the supplemented compared to the placebo group (56.2 ± 18.9 nmol/L *vs*. 30.4 ± 8.6 vs. *p* = 0.029) (Table 3). There were no significant differences in the metabolic health and inflammatory parameters measured

**Table 1:** Participants’ characteristics in Study 1.

	Placebo, N = 4		Vitamin D, N = 4		*p*
	Mean	SD	Mean	SD	
Age (y)	53.8	7.3	52.5	7.5	*0.802*
Height (m)	1.77	0.11	1.69	0.11	*0.344*
Weight (kg)	84.8	13.6	70.5	16.3	*0.211*
BMI (kg/m^2^)	27.0	3.2	24.3	2.8	*0.215*

**Table 2:** Participants’ characteristics in Study 2

	Female, N = 10		Male, N = 10		*p*
	Mean	SD	Mean	SD	
Age (y)	27.2	4.5	30.6	5.0	*0.126*
Height (m)	1.68	0.05	1.81	0.07	*0.002*
Weight (kg)	55.2	5.24	79.9	11.3	*3 × 10^-5^*
BMI (kg/m^2^)	19.5	1.5	24.3	3.1	*0.001*

Values are means and SD; the independent sample *t*-test was used for between group comparison.

**Table 3:** Biochemical markers of subjects in the vitamin D3 supplemented and placebo groups (Study 1). NEFA, non-esterified fatty acids; TG, triglycerides.

	Placebo		Vitamin D		p *
	Mean	SD	Mean	SD	
25(OH)D (mmol/L)	30.4	8.6	56.2	18.9	*0.029*
hsCRP (mg/L)	2.8	3.6	1.0	0.4	*0.368*
IL-6 (pg/mL)	2.2	3.3	0.6	0.1	*0.362*
TNFalpha (pg/mL)	4.0	1.0	3.6	1.1	*0.541*
Glucose (mmol/L)	5.7	0.4	5.6	0.1	*0.555*
Insulin (µU/mL)	3.2	1.8	3.5	2.8	*0.837*
TG(mmol/L)	1.0	0.2	1.4	0.5	*0.164*
NEFA (mmol/L)	0.4	0.2	0.6	0.2	*0.247*
Total cholesterol (mmol/L)	6.4	0.7	5.7	0.5	*0.105*
HDL (mmol/L)	1.3	0.4	1.4	0.4	*0.749*
LDL (mmol/L)	4.5	0.3	3.9	0.3	*0.030*
Adiponectin (µg/mL)	6.0	4.1	5.7	2.2	*0.896*
Leptin (ng/mL)	0.9	0.5	0.7	0.5	*0.617*
Resistin (ng/mL)	3.9	0.6	3.0	1.1	*0.178*
Ferritin (ng/mL)	65.6	39.1	72.0	27.0	0.791

* Unadjusted *p*-value; values are means and SD; the multivariate general linear model (GLM) was used to compare fatty acid (FA) peripheral blood mononuclear cells (PBMC) levels between the vitamin D supplemented and placebo groups.

The most abundant FA in the cells were as follows (given in descending order): C20:4n6, C18:0, C16:0 and C18:1n9c and comprised around 85% of the total FA present in the PBMC cell. There was no significant difference in the FA in the supplemented group compared to the placebo group (Table 4).

**Table 4:** Fatty acids PBMC profile in vitamin D and placebo groups (Study 1).

Fatty Acid (%)	Placebo		Vitamin D		p *	*q*
	Mean	SD	Mean	SD		
C16:0 (palmitic acid)	18.3	2.2	19.5	1.0	*0.373*	*0.667*
C18:0 (stearic acid)	23.6	2.2	22.1	0.5	*0.258*	*0.667*
C18:1n9c (oleic acid-cis)	13.1	1.3	13.2	1.2	*0.880*	*0.667*
C18:1n9t (elaidic acid)	1.6	0.3	1.5	0.2	*0.918*	*0.693*
C18:2n6 (linoleic acid)	7.7	1.5	7.3	0.8	*0.604*	*0.693*
C20:4n6 (arachidonic acid)	30.6	3.0	30.7	2.4	*0.949*	*0.693*
C20:3n6 (osatrienoic acid)	1.7	0.1	2.0	0.3	*0.149*	*0.667*
C20:0 (arachidic acid)	1.1	0.1	1.0	0.1	*0.254*	*0.684*
C22:0 (behenic acid)	1.0	0.3	1.1	0.1	*0.313*	*0.684*
C24:1 (nervonic acid)	0.9	0.1	1.0	0.3	*0.220*	*0.667*
C24:0 (lignoceric acid)	0.5	0.0	0.5	0.1	*0.740*	*0.725*
SFA	44.5	3.9	44.3	1.3	*0.933*	*0.745*
MUFA	15.5	1.2	15.7	1.6	*0.773*	*0.667*
PUFA	40.0	3.9	39.9	2.0	*0.963*	*0.693*

* Unadjusted *p*-value; values are means and SD; multivariate GLM was used to compare FA PBMC levels between the vitamin D supplemented and placebo groups. SFA refers to total saturated fatty acids. MUFA refers to total mono-unsaturated fatty acids, and PUFA refers to total poly-unsaturated fatty acids.

### 2.2. Correlations between Fatty Acids and Metabolic Markers (Study 1)

Examination of the relationship between FA levels and metabolic markers revealed several significant correlations (Supplementary Table 1). There was a strong positive association between C18:0 and hsCRP, IL-6 and adiponectin. A significant positive correlation was also observed between total SFA and CRP and adiponectin, C24:0 and leptin, C20:0 and resistin, Negative correlations were observed between C16:0 and total cholesterol, C18:2n6 and adiponectin, C20:3n6 and resistin, C20:0 and 25(OH)D.

### 2.3. Examination of Fatty Acids and Amino Acids Levels in PBMCs (Study 2)

In the second study, 20 subjects were recruited in order to assess if the PBMC metabolic profile differs between genders. No difference in age between male and female participants was observed in the second study. However, females had significantly lower BMI (19.5 ± 1.5 kg/m^2^) compared to males (24.3 ± 3.1 kg/m^2^) (*p* = 0.001) (Table 2). 

A total of 11 FA present in PBMC were identified and quantified (Table 5). After controlling for BMI, there was a significant difference observed for C16:0 (*p* = 0.025), C18:1n9c (*p* = 0.018) and total MUFA (*p* = 0.015) between males and females. However, using a false discovery rate(FDR )at 5% revealed that none of these were significantly different between the genders. In addition, 10 amino acids (AA) were profiled and quantified (Table 6). In the unadjusted model, the percentage of isoleucine (*p* = 0.003) was significantly higher in males *vs*. females. Aspartic acid was lower in the PBMC from males (*p* = 0.02). After adjustment for BMI, no significant differences were observed between the groups, suggesting a confounding effect of BMI.

In subsequent analysis by BMI groups (low *vs*. high), valine (2.1 ± 0.5 *vs*. 3.1 ± 0.7%, *p* = 0.02) and isoleucine (2.1 ± 0.4 *vs*. 2.7 ± 0.4%, *p* = 0.011) were lower, and aspartic acid (11.3 ± 2.3 *vs*. 8.0 ± 2.0% *p* = 0.003) was significantly higher in the lower BMI group (BMI ≤ 20.6 kg/m^2^). There was no significant difference in the FA levels between the BMI groups.

**Table 5:** Fatty acids profile of PBMC in female and male subjects (Study 2).

w/w (%)	Female		Male		p *	p **	*q*
	Mean	SD	Mean	SD			
C16:0 (palmitic acid)	23.9	2.4	19.6	4.0	*0.008*	*0.039*	*0.096*
C18:0 (stearic acid)	26.9	4.5	23.3	7.0	*0.189*	*0.373*	*0.268*
C18:1n9c (oleic acid-cis)	12.7	1.0	12.7	2.0	*0.998*	*0.020*	*0.849*
C18:1n9t (elaidic acid)	2.7	1.2	2.0	1.6	*0.314*	*0.351*	*0.363*
C18:2n6 (linoleic acid)	3.6	1.2	4.2	1.0	*0.291*	*0.358*	*0.363*
C20:4n6 (arachidonic acid)	25.5	6.3	33.0	10.7	*0.072*	*0.090*	*0.191*
C20:3n6 (osatrienoic acid)	1.6	0.8	1.3	0.4	*0.379*	*0.721*	*0.399*
C20:0 (arachidic acid)	0.5	0.1	0.6	0.1	*0.061*	*0.172*	*0.191*
C22:0 (behenic acid)	1.2	0.2	1.7	0.6	*0.015*	*0.274*	*0.096*
C24:1 (nervonic acid)	0.7	0.2	0.7	0.2	*0.932*	*0.669*	*0.849*
C24:0 (lignoceric acid)	0.7	0.3	0.8	0.3	*0.161*	*0.771*	*0.268*
SFA	53.2	6.3	46.1	10.1	*0.075*	*0.172*	*0.191*
MUFA	16.1	1.7	15.4	2.3	*0.486*	*0.020*	*0.477*
PUFA	30.7	7.8	38.5	11.3	*0.090*	*0.106*	*0.191*

**Table 6:** Amino acids profile of PBMC in female and male subjects (Study 2).

w/w (%)	Female		Male		p *	p **	*q*
	Mean	SD	Mean	SD			
Alanine	8.8	1.4	9.4	1.7	*0.366*	*0.449*	*0.732*
Valine	2.3	0.5	2.9	0.8	*0.034*	*0.694*	*0.117*
Leucine	1.6	0.6	1.9	0.6	*0.247*	*0.596*	*0.62*
Isoleucine	2.1	0.4	2.7	0.4	*0.004*	*0.175*	*0.04*
Glycine	6.1	1.9	6.6	1.2	*0.477*	*0.634*	*0.792*
Serine	3.5	0.5	3.5	0.8	*0.995*	*0.929*	*0.994*
Threonine	1.2	0.3	1.2	0.3	*0.939*	*0.678*	*0.994*
Proline	50.2	4.0	49.9	4.3	*0.872*	*0.866*	*0.994*
Aspartic acid	11.0	2.6	8.2	2.1	*0.017*	*0.578*	*0.085*
Glutamine	13.3	2.5	13.6	1.4	*0.723*	*0.621*	*0.994*

* Unadjusted *p*-value. ** Model adjusted for BMI; values are means and SD; the multivariate GLM was used to compare PBMC metabolites levels between the genders. SFA refers to total saturated fatty acids. MUFA refers to total mono-unsaturated fatty acids, and PUFA refers to total poly-unsaturated fatty acids.

### 2.4. Discussion

Transcriptomic analysis of PBMCs has been successfully implemented in nutrition research [17]. To date, there has been relatively few examples of metabolomic applications using PBMCs. However, this potential application holds great promise and may in fact yield more useful biological information with respect to inflammatory processes compared to analysis of serum or plasma. Moreover, the important interplay between metabolism and immunology has recently come to light [18]. Application of the approach developed here could greatly enhance the interpretation of metabolic responses in immune cells. In the present study, there were no differences in the FA profile of PBMCs in the vitamin D supplemented group compared to a placebo group. However, several of the FAs were significantly correlated with biochemical markers. In the second part of the study, the metabolic profile of the PBMC was expanded to AA, and comparison across genders revealed BMI-, but not gender-, specific AA.

Proportions of FA identified in the PBMC were comparable to the results obtained in other studies [10,19] and closely reproduced the proportions of FA found in the liver in another study [20]. As expected, the most abundant was arachidonic acid (20:4n6), a main energy source for activated immunologic cells that plays an important role in the control of cellular metabolism during inflammation [21,22].

Since the discovery of VDR in macrophages, dendritic cells and activated T and B lymphocytes, vitamin D has been suggested to play a role in modulating immune response [15]. Therefore, we hypothesized in the first study that higher serum total 25(OH)D levels would be accompanied by some changes in the FA profile of the PBMC. The vitamin D group successfully increased their vitamin D status after four weeks of vitamin D3 supplementation, as compared to the control group not receiving vitamin D. However, similarly to the biochemical markers, no significant changes in the FA profile of the PBMC were observed, presumably due to the short length of the intervention. Nevertheless, some significant correlations were observed between FA PBMC content and metabolic biomarkers. Two inflammatory markers, hsCRP and IL-6, had a strong positive association with stearic acid (C18:0), but not palmitic acid (C16:0). Generally, exposure to palmitic acid is associated with its proinflammatory effect on variety of cells [23,24]. It has also been previously reported that C18:0 from PBMC phospholipids was negatively correlated with IL-6 production after inflammatory stimulus [10]. In our study, the results of the association of PBMC FA content with plasma inflammatory markers showed the opposite, but this was observed based on non-stimulated PBMC metabolome and plasma biomarkers levels in healthy subjects. In addition, two other individual SFAs had a positive association with resistin and leptin, which are known as proinflammatory adipokines [25,26]. SFAs possess more proinflammatory functions than unsaturated FA [27]. Adiponectin, in contrast, regarded as an anti-inflammatory adipokine that is generally reduced in obesity [28], was also positively related to the C18:0 and SFA of PBMC. However, a recent study found that adiponectin can induce proinflammatory functions of isolated macrophages and T-cells [29]. Nevertheless, since we did not observe in this study any differences in serum inflammatory markers, as well as the arachidonic acid content of PBMC, we cannot extrapolate the above findings to inflammatory functions following increased vitamin D intake. The release of arachidonic acid followed by the eicosanoid production pathway is the main characteristic of inflammation processes [30].

In the second study, we employed an additional group of younger adults to see if it is possible to expand the profiling of PBMCs to include AA profiling, as well as to test the difference in the metabolite profile of PBMC between genders. Females and males differ in adipose tissue content and distribution, as well as body protein stores [31], which could provoke different FA and AA footprints of the PBMC. However, after controlling for multiple comparisons, only isoleucine significantly differed between genders. Because there was a significant difference in BMI between the genders, we subsequently controlled the statistical analyses for BMI and observed that there were no significant difference between the groups. To our knowledge, this is the first report of the AA content in PBMC. The role of different AAs in cells has been extensively studied. While glutamine plays a major role in the metabolism of immune system cells [32], other AAs have important regulatory functions by affecting protein synthesis, the production of cytokines and other immune functions. Aspartic acid plays an important role in the production of NO through ciruline recycling and is crucial for cell proliferation in response to immunological challenges. Branched Chain Amino acids (BCAAs), including valine and isoleucine, are the essential AA involved in protein synthesis through their action on the mTOR signalling pathway [33] and were found in the second study to be higher in the PBMCs of persons with higher BMI or confounded by BMI in the between genders comparison. Indeed, BCAAs have been shown to be elevated in plasma of obese subjects and contribute to obesity-induced insulin resistance [34].

The present studies have limitations and strengths. The major strength of our study was that the metabolic profiling of PBMCs was combined with extensive blood biochemical analysis, which could give a broad overview of PBMC function in response to FA metabolism and inflammation. In the second study, for the first time, both aqueous (AA) and organic (FA) extracts were analysed, illustrating the overall metabolic profile of PBMCs in a steady, fasting condition, and highlighting the possible differences between BMI rank. The limiting factors include low numbers of subjects; however, this study was set in order to prove the application of PBMC in metabolomic research and not for hypothesis-based purposes. Due to the low number of subjects and the limited power of the study to detect significant differences, the results have to be interpreted with caution. Another limitation of the study is the fact that the PBMC cells include many cell types with different properties regarding their function. Nonetheless, the present study is an important demonstration of the feasibility of applying metabolomics analysis to PBMCs. Further work could be directed at applying this methodology to different isolated blood cell types, which would make biological interpretation of the results easier.

## 3. Experimental section

### 3.1. Study Design and Blood Collection

The protocol was approved by the Human Research Ethics Committee at University College Dublin. Written informed consent was obtained from all subjects according to the guidelines specified in the Declaration of Helsinki. This research was a part of a double blind placebo-controlled study registered as a clinical trial (LS-10-152-Brennan-Gibney, ISRCTN16158244). In the first study (Study 1), eight healthy males and females aged 40–65 years were randomly selected for this study from the subjects receiving either 15 µg of vitamin D3 or placebo in a capsule form for 4 weeks, as described elsewhere [35]. All volunteers continued their usual diets, and no changes in the lifestyle were requested. Several biochemical markers were measured in serum or plasma, depending on the instructions and have been reported elsewhere, including tumour necrosis factor α (TNF-α), ferritin, interleukin-6 (IL-6), insulin, leptin, resistin, hsC-reactive protein (hsCRP), glucose, adiponectin, TG, non-esterified fatty acids (NEFA) and total, LDL and HDL cholesterol. Serum 25(OH)D levels were computed by adding measured serum 25(OH)D2 and D3 levels. In the first study, samples were obtained post the 4-week intervention, and the placebo group was the control group not receiving vitamin D supplementation.

In the second study (Study 2), we aimed to further explore PBMC characteristics by adapting a protocol to measure the amino acid profile of PBMC. For this reason, an additional recruitment of 10 male and 10 female free-living subjects was performed. The subjects were recruited on the University College Dublin campus by use of poster and email advertisement. All volunteers continued their usual diets, and no changes in the lifestyle were requested. The characteristics of the volunteers are shown in Table 1a and b.

Body weight and height were measured, and BMI was calculated: BMI = weight (kg)/height^2^ (m^2^). All of the study subjects were asked to fast for 12 h before blood collection.

### 3.2. Cell Preparation and Extraction of Metabolites

Blood was collected by a trained phlebotomist into BD vacutainer Cell Preparation Tubes (CPT;BD, USA) and processed according to the manufacturer’s instructions. Briefly, samples were inverted 8–10 times, kept upright at room temperature (RT) and centrifuged within 20 min of collection at 2,300 rpm. The PBMC cell layer was then collected into a 15-mL conical tube and washed twice with sterile phosphate buffered saline (PBS) (Invitrogen, New Zealand). The cell pallet was kept at −80^o^C until subsequent analysis.

Cell metabolites were extracted and deproteinised using methanol/chloroform/water method. Cell pellets were resuspended in 1 mL of ice-cold methanol/chloroform mixture (2:1) and kept on ice for 2 min until 40 µL of an antioxidant, butylated hydroxytoluene (BHT), and 300 µL of ice cold water and 300 µL of chloroform were added. The samples were then centrifuged for 5 min at 2,000 rpm, and the organic and aqueous phases were collected.

The lipid extracts were dried under nitrogen, and methyl esters were derivatised immediately after extraction. Extracts were derivatised by methylation using methanolic boron trifluoride (BF3, 14% in methanol). Briefly, to the dry lipid extract, 1 mL of methanol, 1 mL of BF3 methanol and 40 µL of BHT were added. Heptadecanoic acid (C17:0) was used as an internal standard, and 20 µL of the C17:0 (2 mg/mL in methanol) was also added. The samples were boiled for 20 min and methyl esters were extracted by the addition of 2 mL hexane and 2 mL deionised water. The supernatant was collected following centrifugation for 5 min at 2,000 rpm, and the extraction step was repeated. The supernatant was dried under nitrogen and dissolved in 200 µL of hexane for gas chromatography mass spectrometry (GC-MS) analysis.

The aqueous extracts were analysed only in the second study: extracts were dried under nitrogen and kept at −20 °C until analysis. The dried sample was resuspended in 200 µL of water, and 20 µL of the internal standard, ^13^C myristic acid (1 mg/mL in methanol), were added. The sample was then dried under nitrogen and methoximised using 60 µL methoxyamine (20 mg/mL in pyridine) for 17 h at RT. N-methyl-N-trimethylsilyltrifluoroacetamide with 1% trimethylchlorosilane (MSTFA + 1% TMCS) (60 µL) was then added and incubated for 1 h at RT. The samples were dissolved in 210 µL of hexane for GC-MS analysis.

### 3.3. Metabolite Analysis

An Agilent 7890A GC coupled with a 5975C MS with an Agilent HP-5ms 30 m × 250 uM × 0.25 µM column was used to identify organic and aqueous metabolites.

For the organic extracts, 1 µL of sample was injected into the column. Helium was used as a carrier gas with a flow of 1.2 mL/min. The initial oven temperature was 70 ºC, raised to 220 ºC at 5 ºC/min, held for 20 min and then raised to 320 °C at 20 °C/min.

For the aqueous compounds, 1 µL of sample was injected to the column. The initial oven temperature was 70 °C, raised to 320 °C at 5 °C/min, held for 20 min and then raised to 320 °C at 20 °C/min with the transfer line temperature of 250 °C. Helium was used as the carrier gas with a column flow of 1.2 mL/min.

Chromatogram peaks were identified on AMDIS version 2.65 using the National Institute of Standards and Technology (NIST) Library 2.0 (2005). Fatty Acid Methyl Esters (FAME) 37 compound mix (Supelco, Bellefonte, PA, USA) and amino acids (AA) standard (Sigma Aldrich, Saint Louis, MO, USA) were used as known standards for FA and AA, respectively. One quant mass for each peak was specified as a target, and three masses were selected as qualifiers. Metabolites present in the samples were then automatically detected based on retention times and fragmentation patterns and quantified by a comparison of the peak areas calibrated to standard mixtures using an Agilent Chemstation MSD E.02.00.493. FA and AA profiles of PBMC were expressed as the percentage of the total amount.

### 3.4. Statistical Analysis

The independent student *t*-test was used for a comparison of age, weight, height and BMI, between the study groups, as well as for comparison of the metabolite levels between the BMI rank (lower and upper median). Dietary intakes of FA were controlled for energy intake and analysed with general linear model (GLM) ANOVA. GLM ANOVA analysis was employed to compare circulating biochemical markers or the PBMC metabolic profile without adjustment (Study 1) and additionally controlling for BMI (Study 2). The Pearson correlation was used to study the relations between biomarkers measured and the phenotypic characteristics. Statistical analyses were performed using SPSS software for Windows (SPSS v.20, US). An estimate of the false discovery rate (FDR, *q*-value) was calculated to take into account multiple comparisons using the “*q*-value” package (1) in R version 2.15.3. The results were considered significant when *p* ≤ 0.05. Data are presented as the mean and SD.

## 4. Conclusions

In conclusion, stearic acid, but not palmitic acid, correlated positively with inflammatory markers. The work paves the way for future applications of metabolomics in identifying the metabolic profile of blood cells as a measure for dietary intake or physiological status and suggests that there are no major differences in metabolic profiling between genders.

## Supplementary Files

Supplementary File 1 Supplementary Materials (PDF, 83 KB)
