# Supplementary material for: Metabolic Profiling of Human Peripheral Blood Mononuclear Cells: Influence of Vitamin D Status and Gender
Source: Metabolites. 2014 Apr 22;4(2):248–59. doi: 10.3390/metabo4020248 (PMC4101505; doi:10.3390/metabo4020248)
Supplement: Supplementary File 1 — Supplementary Materials (PDF, 83 KB) [file metabolites-04-00248-s001.pdf]

Supplementary Materials

Table S1. Pearson correlations between FA profiles and blood biomarkers (study 1).

		BMI (kg/m2)	25(OH)D (nmol/L)	hsCrp (mg/l)	IL-6 (pg/mL)	Triglycerides (mmol/l)	Total Cholesterol (mmol/l)	Adiponectin (µg/mL)	Resistin (ng/ml)	Leptin (ng/ml)
**C16:0**	Pearson Correlation	–0.133	0.292	0.102	0.070	0.063	**–0.724** *	0.485	0.064	0.314
	p	0.734	0.445	0.794	0.858	0.873	**0.027**	0.186	0.871	0.411
**C 18:2n6**	Pearson Correlation	–0.032	–0.002	–0.562	–0.588	0.092	0.422	**–0.0684** *	–0.135	–0.298
	p	0.935	0.996	0.116	0.096	0.814	0.258	**0.042**	0.729	0.437
**C 18:1n9c**	Pearson Correlation	0.029	0.104	–0.390	–0.302	0.254	–0.278	–0.426	0.006	0.070
	p	0.941	0.791	0.300	0.430	0.510	0.468	0.253	0.988	0.857
**C 18:1n9t**	Pearson Correlation	0.494	–0.045	0.229	0.211	0.343	0.167	–0.104	0.064	–0.416
	p	0.177	0.909	0.554	0.585	0.366	0.667	0.790	0.870	0.265
**C 18:0**	Pearson Correlation	0.651	–0.407	**0.913** **	**0.910** **	–0.159	–0.065	**0.739** *	0.517	0.165
	p	0.058	0.276	**0.001**	**0.001**	0.683	0.867	**0.023**	0.154	0.671
**C 20:4n6**	Pearson Correlation	–0.345	–0.078	–0.294	–0.293	–0.159	0.540	–0.363	–0.284	–0.236
	p	0.363	0.842	0.442	0.444	0.682	0.134	0.336	0.459	0.541
**C 20:3n6**	Pearson Correlation	0.016	0.528	–0.179	–0.260	**0.764** *	–0.186	–0.280	**–0.769** *	–0.143
	p	0.968	0.144	0.646	0.500	**0.017**	0.632	0.465	**0.015**	0.714

Table S1. *Cont.*

		BMI (kg/m2)	25(OH)D (nmol/L)	hsCrp (mg/l)	IL-6 (pg/mL)	Triglycerides (mmol/l)	Total Cholesterol (mmol/l)	Adiponectin (µg/mL)	Resistin (ng/ml)	Leptin (ng/ml)
**C 20:0**	Pearson Correlation	0.386	**–0.717***	0.195	0.306	–0.353	–0.012	0.178	**0.890****	–0.249
	p	0.305	**0.030**	0.615	0.423	0.351	0.976	0.647	**0.001**	0.518
**C 22:0**	Pearson Correlation	–0.310	0.294	0.367	0.393	–0.172	–0.108	0.437	–0.134	0.232
	p	0.417	0.442	0.331	0.296	0.657	0.782	0.239	0.731	0.549
**C 24:1**	Pearson Correlation	–0.462	0.660	–0.156	–0.189	–0.008	–0.621	0.206	–0.132	0.454
	p	0.211	0.053	0.688	0.627	0.984	0.075	0.595	0.735	0.220
**C 24:0**	Pearson Correlation	**–0.697***	**0.709***	–0.170	–0.247	–0.396	–0.083	0.220	–0.329	**0.728***
	p	**0.037**	**0.032**	0.663	0.523	0.291	0.831	0.569	0.388	**0.026**
**SFA**	Pearson Correlation	0.304	–0.063	**0.673***	0.659	–0.092	–0.504	**0.808****	0.381	0.321
	p	0.426	0.871	**0.047**	0.054	0.814	0.166	**0.008**	0.312	0.399
**MUFA**	Pearson Correlation	0.052	0.183	–0.336	–0.0263	0.295	–0.314	–0.379	–0.002	0.052
	p	0.895	0.638	0.377	0.493	0.440	0.411	0.315	0.996	0.894
**PUFA**	Pearson Correlation	–0.303	–0.025	–0.489	–0.505	–0.037	0.610	–0.604	–0.355	–0.330
	p	0.427	0.950	0.182	0.166	0.925	0.081	0.085	0.348	0.386
